# Structure and complexation mechanism of aqueous Zn(II)-acetate complex studied by XAFS and Raman spectroscopies

**DOI:** 10.1007/s44211-024-00549-z

**Published:** 2024-04-05

**Authors:** Alvaro Munoz-Noval, Kazuhiro Fukami, Takuya Kuruma, Shinjiro Hayakawa

**Affiliations:** 1 Department of Materials Physics, Faculty of Physics, University Complutense of Madrid, 28040 Madrid, Spain; 2 Department of Applied Chemistry, Graduate School of Engineering, Hiroshima University, Hiroshima, 739-8527 Japan; 3 Department of Materials Science and Engineering, Kyoto University, Kyoto, 606-8501 Japan; 4 Present Address: Mitsui Mining and Smelting Co Ltd, Shinagawa-Ku, Tokyo, Japan

**Keywords:** Metal chelates, Structure of aqueous complexes, Raman spectroscopy, XAFS in liquids

## Abstract

In this work, the structure of Zn acetate has been determined by a combination of X-ray absorption fine structure and Raman spectroscopy. We have analyzed the local atomic environment and the main vibrational bands of the acetate and Zn acetate at different pH. The results suggest that Zn acetate complex acquires a bidentate structure that modifies its first coordination shell. Meanwhile, the coordination shell of the hydrated Zn cation is formed by 6 hydroxides at a mean distance of 2.06 Å, the coordination shell of the Zn cation in the complex is formed by 2 hydroxides and 2 oxygens from the carboxyl group of the acetate, at a mean Zn–O distance of 1.96 Å. The structure of the Zn acetate complex is compared to those of Zn malonate and Zn citrate, none of which present a reduction in the coordination shell neither a shrinkage of the Zn–O shell distance.

**Graphical abstract:**

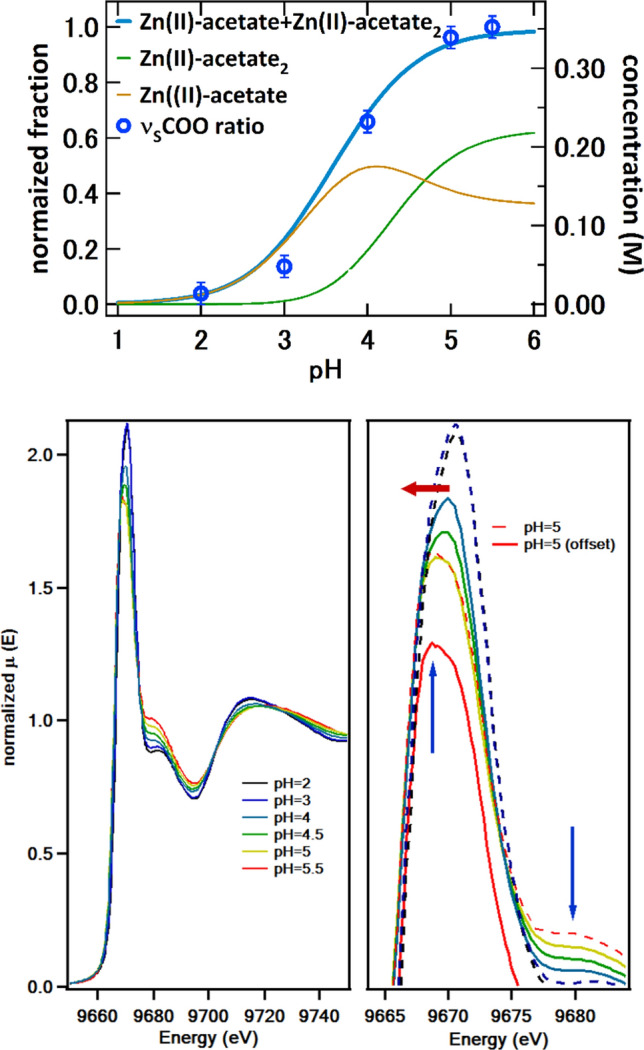

**Supplementary Information:**

The online version contains supplementary material available at 10.1007/s44211-024-00549-z.

## Introduction

The use of chelating agents is nowadays broadly employed in industry for plating processes [1], heavy metal removal from soils, wastewaters and contaminated environments [2–5], etc. Particularly, in electrochemistry, the use of chelating agents has been successfully implanted for improving the metal deposition within nanoporous electrodes [6, 7]. The chelation of metals with carboxylic ligands allows tuning the size, the surface charge, and the solvation of the metal cation. All these physicochemical parameters are tightly involved in the occurrence of the surface-induced phase transition (SIFT) which causes accelerated mass transfer within nanopores under certain conditions. 

In previous works, we have focused on studying which conditions determine SIFT when depositing Zn from Zn(II)-malonate and Zn(II)-citrate complexes [7]. To clarify the mechanism and the conditions required to happen, the relation between chelate structure and its performance in the SIFT, this work aims to study the Zn complex with acetic acid and compare it with more complex carboxylic acid complexes. In the carboxylates, the relation between structure and vibrational properties in acetate interacting with metal ions was studied by Nara et al. employing ab initio methods [8]. They found a relation among the relative frequencies of the vibrational modes of the symmetric (*ν*_s_) and asymmetric (*ν*_a_) stretching of the COO, and the difference of the frequencies, in cm^−1^ unit, was related to the spectrum with main structural parameters, such as the O–C–O angle (${\theta }_{{\text{OCO}}}$) and the relative asymmetry of the C-O bond lengths (${\delta }_{r})$:

1 $${\Delta }_{{\text{s}}-{\text{a}}}=1818.1{\delta }_{r}+16.47\left({\theta }_{{\text{OCO}}}-120\right)+66.8$$

The *∆*_s-a_ value for the ionic state (deprotonated acid) is employed as a reference for determining if a complex is bidentate (*∆*_*s-a*_ lower than reference) or mono-dentate (*∆*_s-a_ significantly larger than reference). This model has been in good agreement with experimental results for Ca [9, 10] or Mg [11] complexes. In the case of Zn, it has been extensively debated about the mono-dentate or bidentate interaction with the acetate ligand [12]. In this work, we have performed a detailed study of the Zn(II)-acetate complexation as a function of the pH, in order to understand the relation between the electrochemical performance of the complex with its structure, by a combined analysis using Raman and XAFS spectroscopies.

Zn complexes are critical in biological processes. Particularly, Zn(II)-acetate is a compound of medical interest [13]. As far as we know, its crystal structure was firstly studied in a di-hydrated, solid form by van Niekerk et al. [14] and later by Clegg et al. [15] and Ishioka et al. [16], using X-ray diffraction. In its solid form, they observed Zn adopts a distorted octahedral structure coordinated by two water O atoms and four acetate O atoms. However, up to our knowledge, the structure and coordination of the complex in its aqueous form has not been yet reported. In this regard, X-ray absorption fine structure (XAFS) spectroscopy, an element-selective, local probe technique that allows to characterize metal–organic compounds even in solution [17, 18] and determine with precision its local environment, including coordination, structure, and first-neighbor distances.

In this work, the structure and complexation of Zn(II)-acetate have been studied by XAFS and supported on Raman measurements to determine the pH-dependent complexation and structure. Finally, a comparative study of similar short-chain Zn(II)-carboxylates has permitted to get further insights into the coordination and structure of zinc carboxylates in an aqueous solution.

## Experimental section

### Materials

All the guaranteed reagents were used without further purification. Zinc sulfate heptahydrate (ZnSO_4_⋅7H_2_O) was purchased from KISHIDA CHEMICAL Co., Ltd., Japan, and acetic acid (AA), malonic acid (MA), and citric acid (CA) were purchased from Nacalai Tesque, INC. Japan. Solutions were prepared by dissolving the reagents in ultra-pure water. Two types of mother solutions were prepared, and they contained one of the carboxylic acids (AA, MA, CA) and Zn of different molar ratios. One contained 1.0 M acetate (Ac) and 0.5 M Zn, and the other contained 2.0 M Ac and 0.5 M Zn. Solutions of different pH values were prepared by adding small volumes of commercial 1.0 M hydrochloric acid or sodium hydroxide.

### Calculations of speciation curves

The Equilibrium speciation curves and fractional speciation diagrams for the aqueous Zn acetate system have been calculated with Visual MinteQ software v.3.1 [19]. The equilibrium constants and Debye Hückel parameters included in the Visual MinteQ database have been employed for the calculations. Only major chemical species in the aqueous solution have been included for calculations. The speciation curves pKas and complex formation constants were calculated with the parameters listed in Table 1. The equilibrium fractional composition diagrams are calculated by determining a few chemical parameters (i.e. dissociation constants, etc.) and assuming thermodynamic relations. All the Zn species are in divalent cations. By increasing the pH the acetic acid is dissociated in ionic acetate and, in presence of the Zn(II) cation, the fraction of Zn(II)-acetate increases progressively in two species, Zn(II)-acetate^+^ or Zn(II)-acetate_2_. It is predicted that the fraction of the Zn(II)-acetate_2_ becomes dominant in the high pH region when the ratio of Ac/Zn is higher.

**Table 1:** Dissociation constant of the carboxylic acids studied

Acid	Ligand (L)	pKa	References
Acetic acid (AA)	Acetate	4.76	[20]
Malonic acid (MA)	Malonate	2.83, 5.69	[21]
Citric acid (CA)	Citrate	3.09, 4.75, 5.41	[22]

### XAFS measurements

XAFS experiments at the Zn K-edge were carried out at BL01 and BL05 at SPring-8 (Hyogo, Japan) in transmission mode. The intensities of the incident and transmitted beams were monitored by ionization chambers filled with N_2_ at atmospheric pressure. The liquid cell had two Kapton windows for the incident and transmitted beams, and the thickness of the cell was adjusted using an appropriate spacer for optimizing absorbance. The typical cell length was set to 3 mm. The spectra of 1.0 M ZnSO_4_ and Zn(II)-acetate aqueous solutions at different pH conditions (i.e. 2, 3, 4, 4.5, 5, and 5.5) were acquired. Additional XAFS measurements in transmission mode were carried out in Zn(II)-malonate and Zn(II)-citrate at different pH: 2, 3, 4, 5 and 6 for Zn(II)-malonate and 1, 1.8, 3 and 4.8 for Zn(II)-citrate 1.0 M solutions.

XAFS data was reduced by employing the software pack Demeter by standard procedures [20]. To properly compare the XAFS spectra of the different solutions analyzed in this work, they were normalized using the same normalization procedure and setting the same normalization parameters. The pre-edge range was set from –200 eV to –30 eV respect to the edge, the normalization range from 110 to 780 eV respect to the edge, setting an order 3 of normalization [23]. EXAFS fittings were performed simultaneously in k and r-spaces employing theoretical paths from crystallographic references calculated with FEFF [21]. Best fits presented correspond to r-space, performed for k intervals from 1.8 to 10 Å^−1^ weighted in k^3^ and r intervals of 1.2 to 3.3 Å. The resolution limit for distinguishing two close shells by EXAFS is directly related to the maximum photoelectron wavenumber reached (that is, the kinetic energy of the photoelectron): $\mathrm{\delta R}=\pi /2{k}_{{\text{max}}}$. For the present work, on average the *k*_max_ is 10 Å^−1^, meaning that two shells must be separated at least by ca. 0.16 Å to be resolved. Ab initio calculations of the structures have been performed employing ab initio structures constructed employing CRYSTALFFREV [22] to obtain adequate FEFF input data files.

### Raman measurements

Raman measurements were performed in a standard Raman spectrometer equipped with a single monochromator (T64000) and a diffraction grating (600 l/mm). The excitation source was an Ar^+^ laser (*λ* = 514.5 nm) with an output power set to 400 mW. Several spectra were acquired for different sets of solutions for checking repeatability. The measured solutions in the Raman experiments were 0.1 M, 1.0 M, and 2.0 M CH_3_ COOH, (AcH) and 2.0 M Zn(II)-acetate, in the same range of pH as in the XAFS experiments. Raman spectra of water were acquired in every experimental session for subtracting the background of AcH and Zn(II)-acetate solutions taken under the same conditions.

## Results and discussion

### Raman spectra of the Zn complexes

**Fig. 1:**
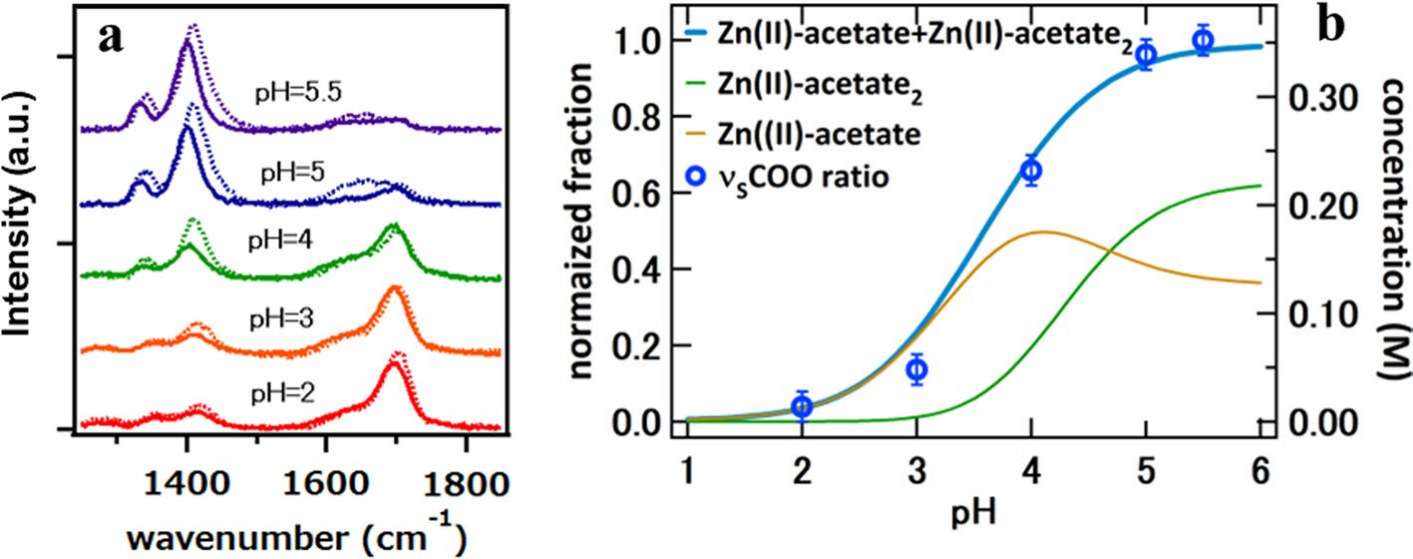
Raman spectroscopy for the 1.0 M Zn(II)-acetate and AA solutions in the region of the C=O, ν_a_COO and ν_s_COO bands: **a** Zn(II)-acetate solutions (dashed lines) with pH ranging from 2 to 5.5 compared to 1.0 M AA solutions (solid lines); **b** dissociation curve of the Zn(II)-acetate 0.5/1.0 M with the intensity ratio of the ν_s_COO bands of the Zn(II)-acetate solution and AA solution with equal pH

In a previous paper, we investigated the complexation of Ca(II)-acetate, and the main bands in the vibrational spectra were identified for this complex based on the literature [10, 23–25]. To study the complexation of the cation, special attention was paid to carbonyl (C=O) and COO symmetrical and asymmetrical stretching vibrational modes (ν_s_COO and ν_a_COO, respectively). The correlation between the intensity of ν_s_COO and C=O bands with the dissociation fraction of the carboxyl at different pH were also identified as useful signs for complexation. The intensity of the C=O band is linked to the protonated carboxyl (Ac-H) whereas the ν_s_COO appears after the dissociation of the acid. The presence of the ν_s_COO band is compatible with an ionic acetate complex or with a bidentate acetate-metal complex when there are metal cations in the solution. The Raman spectra and speciation curves for the solutions of 1.0 M acetic acid (AA) and solutions with ZnSO_4_/AA 0.5/1.0 M, respectively, are included in Fig. 1 at the spectral region where the ν_s_COO band appears (around 1408 cm^−1^) for different pH. Figure 1a shows the Raman spectra in the region of the C=O, ν_a_COO and ν_s_COO bands for the 1.0 M Zn(II)-acetate and AA solutions in the range of pH of study. The spectra show a clear band intensity dependence with the pH in both solutions, AA and Zn/AA.As abovementioned, the relative ν_s_COO band intensity (ν_s_COO ratio) allows to semi-quantify the species concentration and compare with the theoretical speciation curves. In Fig. 1b, this ν_s_COO ratio used as an indicator of the relative proportion of complexated Zn is compared with the speciation curves obtained numerically from thermodynamic calculations. The speciation curves considers two main Zn(II)-acetate species: once complexed Zn(II)-acetate and twice complexed Zn(II)-acetate (Zn(II)-acetate_2_). The total concentration of Zn in form of Zn-complexes (as a sum of both species) in the calculated speciation curve does reach the Zn proportion (0.5 M) in solution because other Zn species in solution have not been included in the figure (see Figure S2 in the Supplementary Info).

The relative shift *(****∆***_s-a_) measured in the Zn(II)-acetate solution at pH 5.5 provide values of about 154 cm^−1^. To determine the character of the Zn(II)-acetate applying Nara’s criteria, we obtain the ***∆***_**s-**a_ value for the ionic state of the acetate (deprotonated) as reference [8, 9] using the band intensity measured in the Raman spectra of Zn(II)-acetate solution at pH 5.5 (Figure S1). The experimental value for the Ac solution with pH 7 (not shown), mainly formed by dissociated acetate (pKa = 3.71) is obtained from its corresponding Raman spectra, resulting on a value of 141.8 cm^−1^. Thus, pointing to an ionic or weak bi-dentate character of the Zn(II)-acetate.

The Raman bands for the protonated acetate (Ac-H at 878 cm^−1^) and the C–C symmetric stretching (ν_s_CC at 915 cm^−1^) are also sensitive to dissociation and complexation. The ν_s_CC band is strongly coupled to the vibrational modes of the carboxyl and is employed to determine the complexated/non-complexated acetate in solution [10]. This band presents a band splitting for large cations ^10^ but is not resolved in the case of Zn. Also, the Ac-H band is sensitive to acid dissociation previous works [26]. It is probably related to out-of-plane deformation of the OH in carboxylic groups. The relative intensities of these two bands are represented with the respective speciation curves of the dissociated acetate, protonated acetate, and Zn(II)-acetate species. Ac-H band and ν_s_CC bands reproduce with high fidelity the slope of the speciation curve at both concentrations (Figure S3). Moreover, the normalized speciation curves of the ionic acetate and Zn(II)-acetate species present a gap which is roughly reproduced by the relative intensity of the ν_s_CC band in the Zn/Ac solutions. Therefore, these two bands are quantitative indicators of the dissociation and complexation of the acetate.

### XAFS analysis of zinc acetate in aqueous solutions

**Fig. 2:**
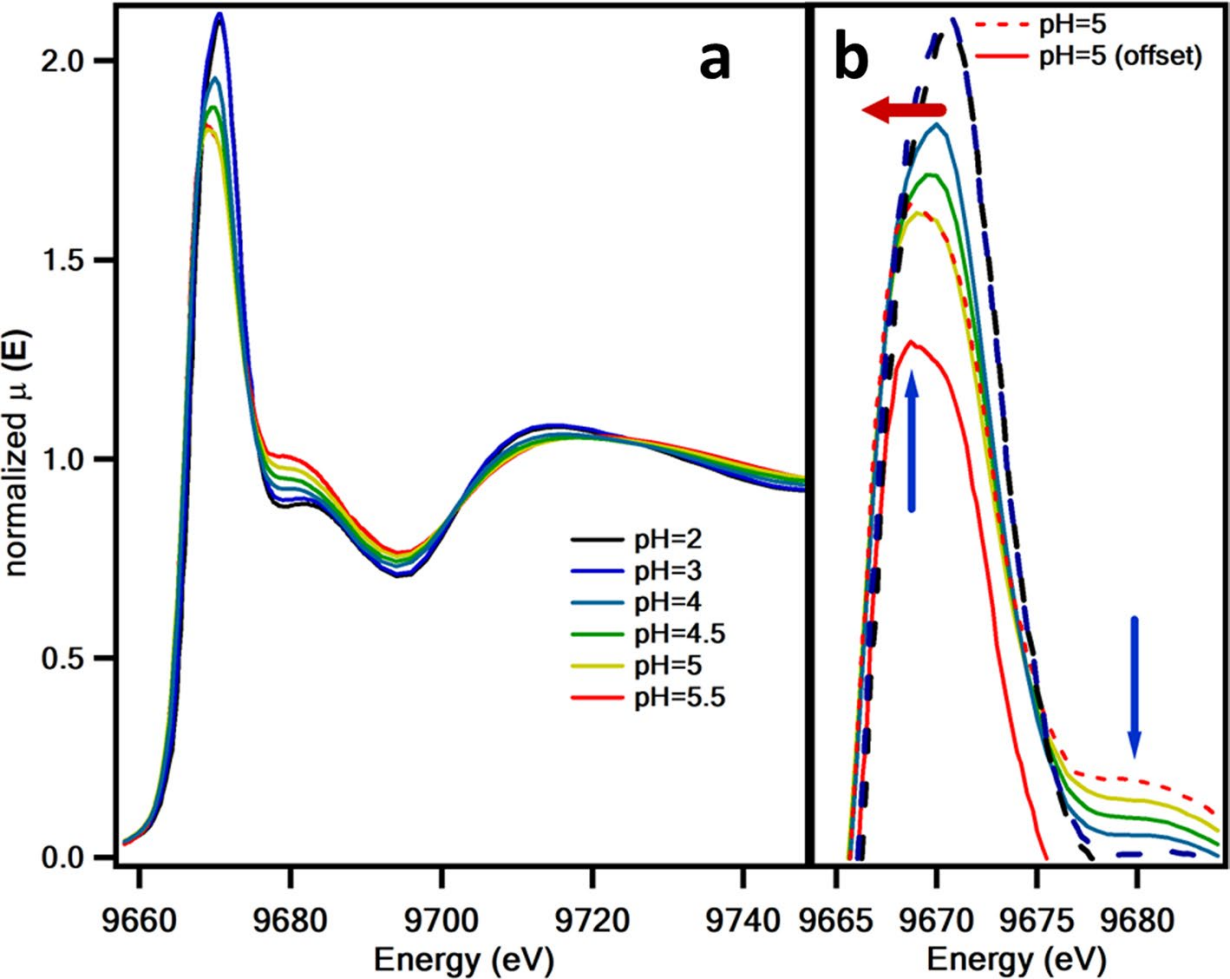
XANES spectra at the Zn K-edge of the Zn/Ac solutions with pH ranging from 2 to 5.5 **a**, showing details of the main peak in **b**

XAFS measurements of Zn solutions with acetic, malonic, and citric acids (Zn(II)-acetate, Zn(II)-malonate and Zn(II)-citrate solutions in the following) at different pH were performed at the Zn K-edge (9659 eV). In Fig. 2, the pH-dependent X-ray absorption near edge spectroscopy (XANES) spectra of the Zn(II)-acetate solutions are depicted. The intensity of the main peak clearly decreases with the increase of pH around the pKa of acetic acid. The peak positions slightly shift to lower energies while the peak profile becomes asymmetric. Three isosbestic points, just before the main peak at about 9673 eV, 9710 eV, and 9725 eV, can be clearly distinguished and indicate the coexistence of two main species contributing to the spectra, presumably hydrated Zn and Zn(II)-acetate. The decrease in the intensity of the main peak with increasing pH upon pKa may evidence the variation of the Zn–O bonding distance or the decrease of the electronegativity of the Zn–O bonding. Additionally, a charge transfer effect with the ligand caused by the covalency causes a slight energy shift for increasing pH [27, 28]. There is a noticeable asymmetry of the main peak at the highest pH, that is probably related to a change in the local symmetry of the Zn. The electronic configuration of Zn, with the 3d level filled (3d^10^4s^2^) is the cause of any change in the edge and pre-edge caused by the local symmetry of the cation would be subtle or practically unobservable [29].

**Fig. 3:**
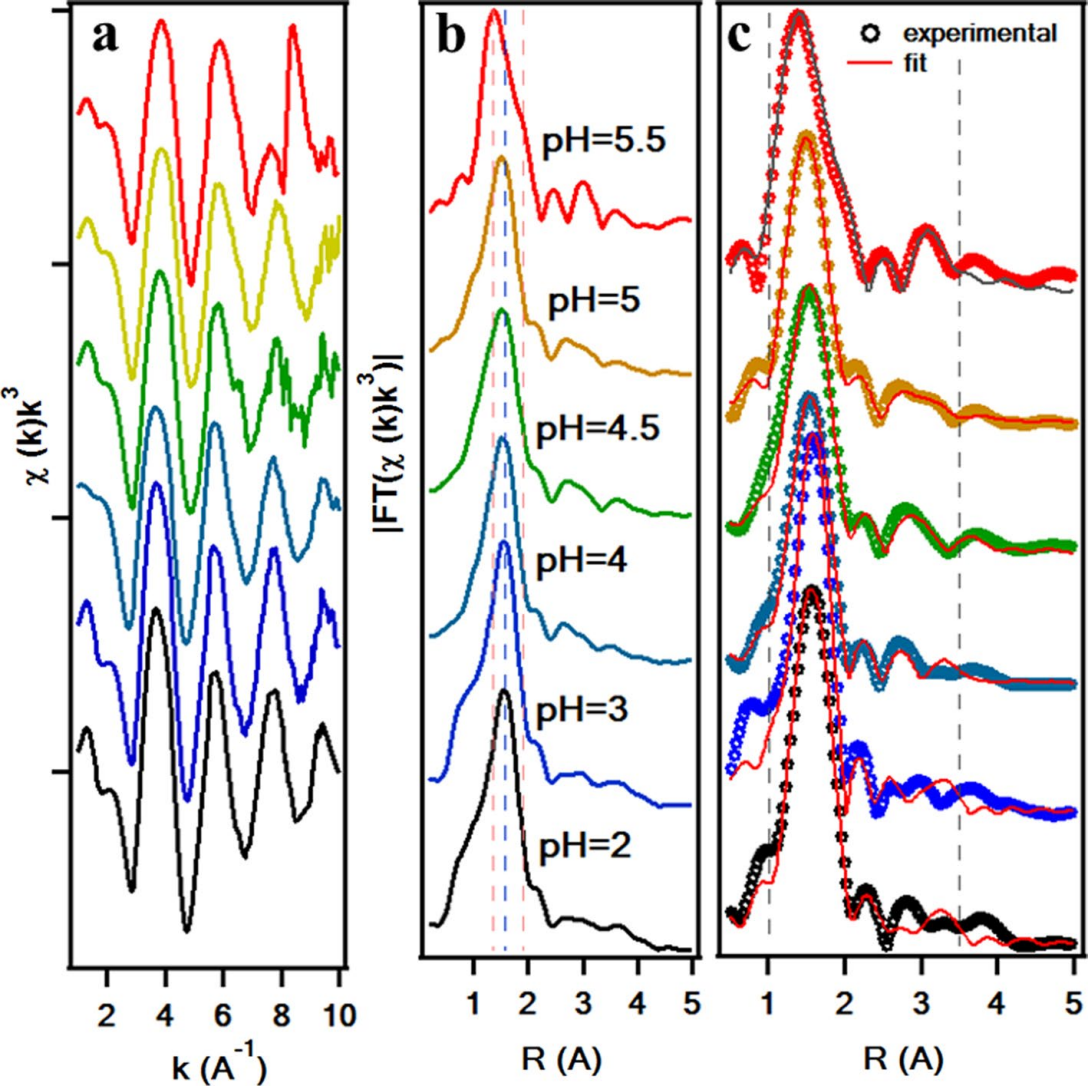
**E**XAFS spectra of the Zn(II)-acetate solutions: **a** raw EXAFS of the Zn(II)-acetate solutions with pH ranging between 2 and 5.5; **b** FT of the EXAFS data included in **a**; **c** FT of the EXAFS spectra and fittings of **b**

The Extended X-ray absorption fine structure (EXAFS) data of the Zn(II)-acetate solutions and their corresponding FT are included in Fig. 3 a and b, respectively. The main peak observed at about 2.06 Å (ca. 1.8 Å in the figure, there is no photoelectron phase correction) corresponds to the Zn–O first shell. A second region with a less intense signal between 2 and 4 Å is due to multiple scattering processes. The first coordination shell of the Zn in aqueous solutions is formed by 6 Oxygens from the hydration sphere in octahedral coordination [30–32]. The multiple scattering signal is related to the multiple collisions of the photoelectron with the atoms in the proximities, including outer hydration shells, anions, etc. The multiple scattering signal shape changes noticeably when increasing pH conditions upon the pKa of the acetate (above pH 5). The EXAFS has been fitted to get the local structure parameters (Table 2). The EXAFS fitting model for the Zn(II)-acetate (Fig. 3c) is a structure with hydrated Zn ions in octahedral coordination.

**Table 2:** Structural EXAFS parameters obtained from the fittings in the Zn(II)-acetate solutions

	pH	2	3	4	4.5	5	5.5
	EXAFS						
O	N (O)	6.0 (5)	6.7 (5)	6.3 (5)	6.7 (5)	5.9 (5)	4.2 (5) 2.4 (5)
	R(Zn-O1)	2.08 (1)	2.05 (2)	2.06 (2)	2.04 (2)	2.02 (2)	1.96 (2)
	R(Zn-O2)				–	–	2.74 (2)
	σ^2^ (O1) σ^2^ (O2)	0.011 (2)	0.012 (1)	0.010 (2)	0.012 (2)	0.011 (1)	0.0033 (7) 0.0133 (8)
	σ^2^ (MS)	0.012 (1)	0.018 (3)	0.017 (2)	0.015 (3)	0.009 (2)	0.015 (2)
	E	2 (1)	5 (1)	4 (1)	6 (1)	5 (1)	7 (1)
	R-coef	0.010	0.011	0.004	0.005	0.003	0.005
	χ^2^	100	166	210	302	156	170

Remarkably, the Zn–O shell shifts to lower distances, of about 0.1 Å in the case of pH above the pKa, and the best fit is obtained with a first coordination shell of 4.2(5) oxygens at 1.96(2) Å including a second coordination Zn–O shell with average coordination of 2.4 (5) at 2.74(2) Å (Table 2).

### A comparative XAFS study of zinc carboxylates

**Fig. 4:**
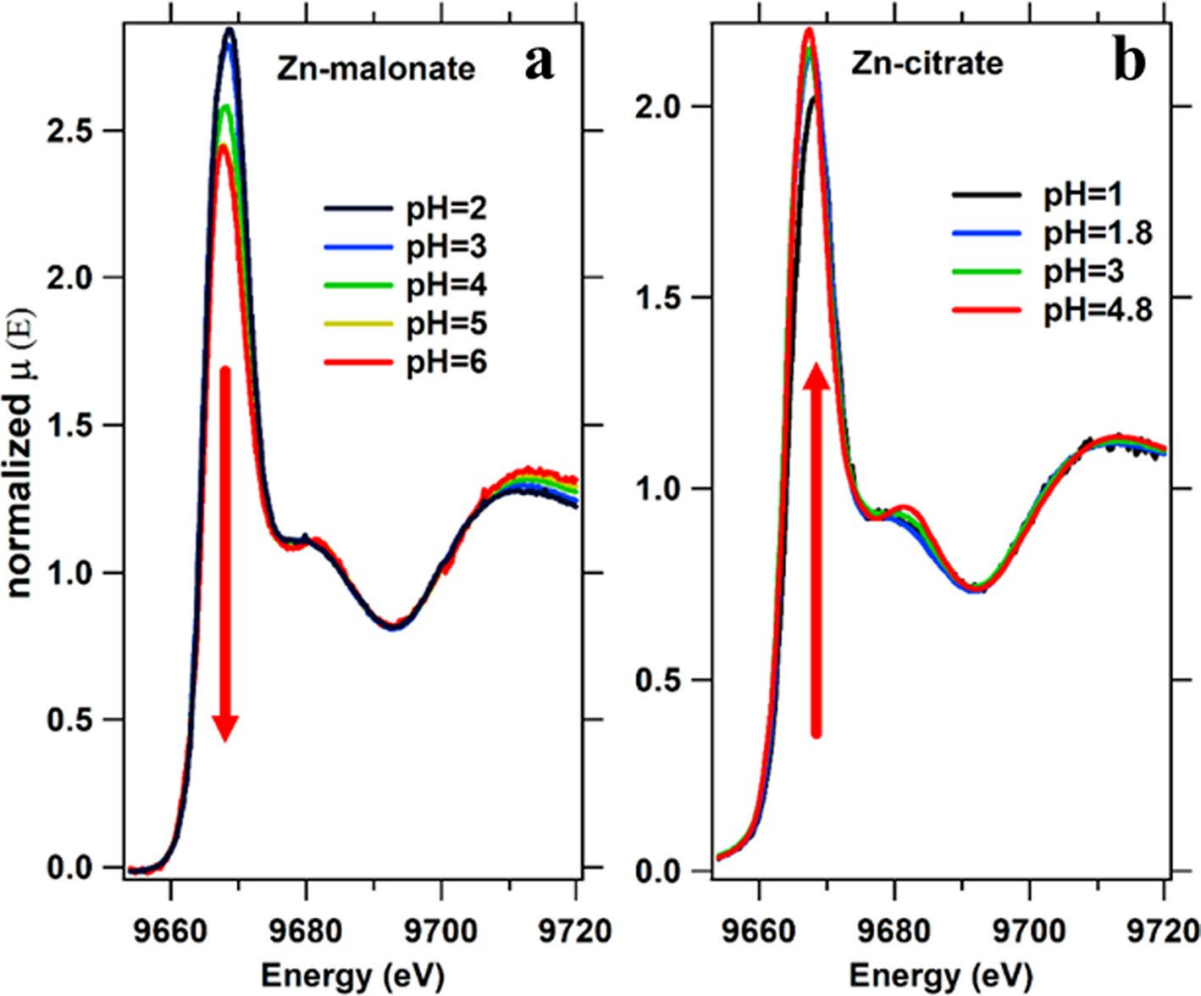
XANES of the Zn(II)-malonate and Zn(II)-citrate: spectra at the Zn K-edge of the **a** Zn(II)-malonate solutions with pH ranging from 2 to 6 and **b** Zn(II)-citrate ranging from pH 1 to 4.8

To further understand the complexation mechanism and the complex structure, similar experiments have been conducted on Zn solutions with malonate and citrate salts (Zn(II)-malonate and Zn(II)-citrate, respectively) at different pH within the range of the first and second pKa [7]. In Fig. 4, the XANES spectra of both groups of solutions at different pH are shown. The intensity of the main peak of the Zn(II)-malonate solutions spectra decreases with increasing pH as there is a slight shift of the main peak center to lower energies. These two features were observed also for the Zn(II)-acetate solutions upon pH increase, as well as an increase of the second resonance intensity and deformation of the main peak shape at pH above pKa. In the case of Zn(II)-malonate solutions, the trend is equivalent at pH > 4, and the spectra do not change for pH 5 and pH 6. In the group of Zn(II)-citrate, solutions the effect of pH in the main peak intensity is the opposite, as it increases with pH. The decrease of the Zn–O covalent bonding upon complexation with malonate could explain similar behavior of the XANES main peak in Zn(II)-acetate, as the lowest peak intensity is observed at pH 4 (first pKa of the malonic acid). The evolution of the spectra with pH for the Zn(II)-citrate solutions behaves the opposite the main peak intensity increases with pH, indicating an increase in the Zn–O bonding.

**Fig. 5:**
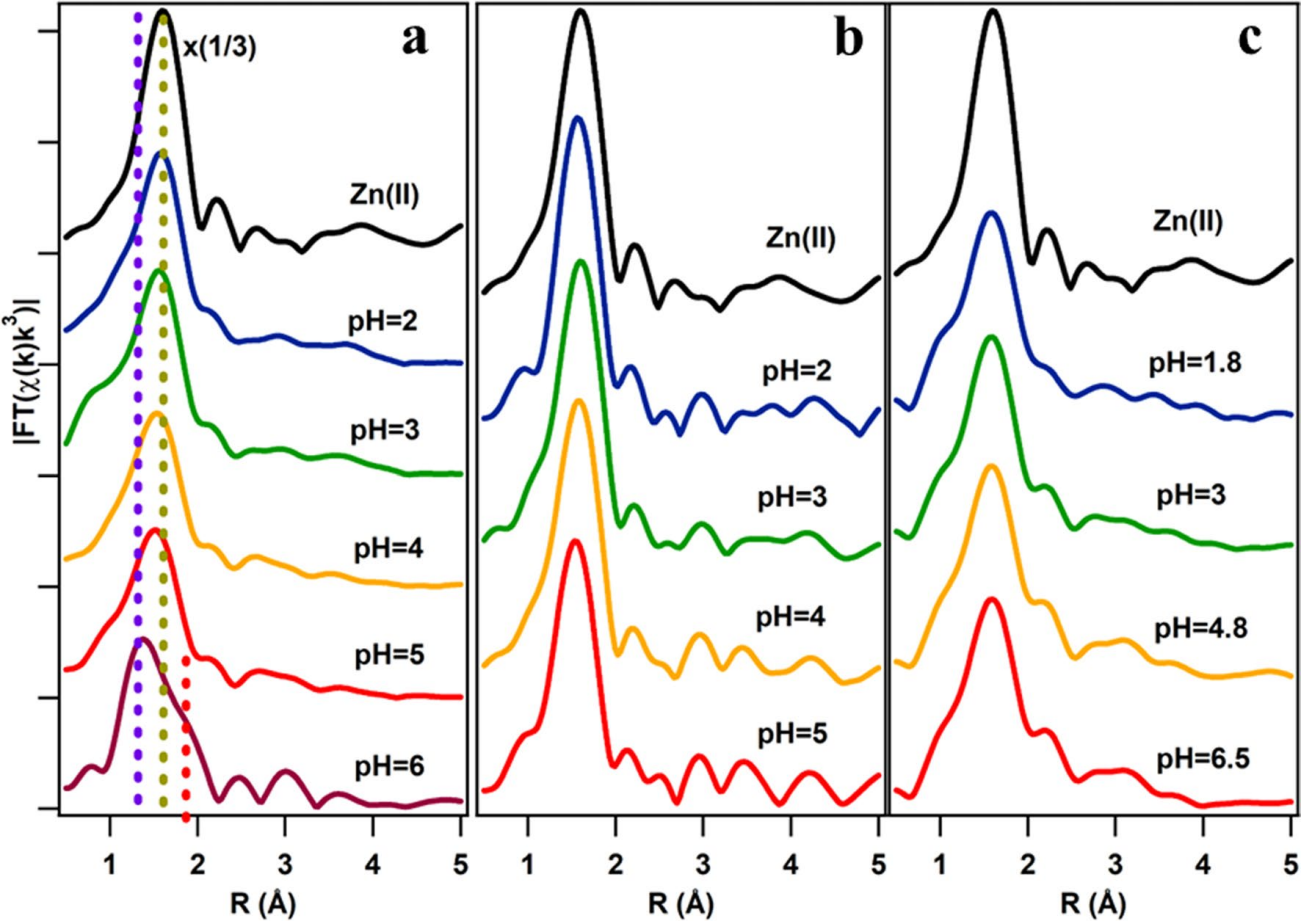
FT of the EXAFS of the Zn carboxylate solutions: **a** Zn(II)-acetate, **b** Zn(II)-malonate and **c** Zn(II)-citrate at different pH conditions. The FT intensity of the Zn(II) solution in **a** is multiplied by a factor 1/3 to be compared with the spectra of Zn(II)-acetate solutions. In **a**, the dashed lines helps the reader to identify the evolution of the main features of the EXAFS signal with increasing pH

The FTs of the EXAFS spectra in the Mal(Cit)/Zn solutions at different pH are summarized in Fig. 5. The FTs of the EXAFS signal show similar features in the three Zn complexes (Ac/Mal/Cit): the main peak at ca. 2 A and a multiple scattering signal above 2.5 A. A change in the MS region at pH above the respective pKa is observed in the three groups of solutions, coinciding with complex formation. The fittings results of the EXAFS spectra of each complex at the pH > pKa condition are summarized in Table 3. There are two main differences between the FT of the EXAFS of the three Zn complexes. First, in the average coordination of the first atomic shell and second, in the first shell distance. Meanwhile, in Zn(II)-acetate and Zn(II)-malonate the first shell is on average formed by 4–5 oxygen atoms, in the Zn(II)-citrate the average number of atoms in the shell is almost 6, as in the case of hydrated Zn(II). The Zn–O shell distance in the Zn(II)-citrate is considerably larger than for the Zn(II)-acetate and Zn(II)-malonate (1.96 Å), and closer to the Zn(II) in solutions (2.06 Å), in opposition to the Zn(II)-acetate and Zn(II)-citrate. The interplay of both parameters, average shell coordination, and distance, would be related to the observed evolution in the XANES spectra. In the case of Zn(II)-acetate/Zn(II)-malonate, there is a diminishing in the main peak intensity, competing with a shell radius decrease that by itself would lead to an increase of the main peak intensity. However, the interplay of these two parameters and the decrease in the coordination of the first shell, observed by EXAFS, may result in the observed XANES features. The opposite reasoning would apply for Zn(II)-citrate because the shell coordination and radius are very close to the hydrated Zn, the peak intensity increase should be related to an increase in the covalency of the Zn binding.

**Table 3:** EXAFS parameters of the best fit in the Zn(II) aqueous solution of the different complex studied

Zn(II)-	1st Zn–O shell			2nd Zn–O shell		
	N	R(Å)	DW	N	R	DW
Hydrated	6.0 (5)	2.06 (2)	0.011 (1)			
Acetate	4.2 (5)	1.96 (2)	0.0033 (5)	2.4 (5)	2.74 (2)	0.013 (2)
Malonate	4.8 (5)	1.96 (2)	0.014 (1)			
Citrate	5.9 (5)	2.02 (2)	0.013 (1)			

At pH 5.5, the coordination of the Zn–O shell distance contracts to 1.96 Å, and its coordination decreases to 4. The best fit is performed including a second shell (Zn–O) with average coordination 2 at 2.74 Å. Considering the Raman data, the most plausible structure of the Zn(II)-acetate complex is a weak bidentate (some authors use the term ionic) structure where the hydration sphere consists of a 4 O and the acetate O is placed at a second shell [33]. Several authors [16, 34] have found evidence that Zn(II)-acetate does not follow Nara criteria when has a chelating structure or low symmetry bridging or monodentate. However, following Ishioka, the rocking modes in the spectra may contain additional clues on the character and structure of the complex. We have preliminary compared the O-C-O out-of-plane rocking modes of the acetate upon dissociation with the corresponding to the complexation with Zn cations. The data presented as supplementary information (Figure S4), can be compared to the spectra measured in equivalent Ca(II)-acetate solutions [10], showing noticeable differences. For instance, the rocking band in the Zn(II)-acetate shifts clearly to lower frequencies as the dissociated species ratio increases (about 30 cm^−1^). A similar behavior has been observed for the Ca(II)-acetate [10], but presenting larger shift (above 50 cm^−1^). Such a change in the mode frequency is compatible with a change in the spring force.

The Zn(II)-acetate spectrum shows a more complex behavior, being the band split in two above the pKa and its intensity noticeably damped with respect to the bidentate Ca(II)-acetate and ionic species [35–41]. These Raman spectral features and the EXAFS results may be compatible with the formation of the Zn(II)-acetate_2_ complex at enough high pH conditions. From our experience, such Zn(II)-acetate_2_ complexation is possible and relatively stable. In such structure, the first coordination shell may be formed by Zn–O of the acetate group and the second by the solvation sphere. We have observed similar behaviour in other Cu(II)-lactate complexes in alkaline solutions [42]. In this work, the complexation of Cu^2+^ with lactate showed a very slow complexation. This means that the fraction of the species may change with time after preparation and the deviation is prominent just near the neutralization point at an alkaline side. This suggests that in the Zn(II)-acetate solutions near pH 5 the fraction of the species in the solution may fluctuate, even in equivalently-aged samples. For the EXAFS experiments, we worked with relatively fresh samples, prepared with few hours in advance (we fabricated Zn/AA solutions of a maximum pH 6, but even at these pH values, we did not observe any precipitation). For enough large reaction times after sample preparation, the EXAFS at pH 5 may present signatures of Zn(II)-acetate_2_ formation, but it would be the subject of a future work on the complex titration and complexation kinetics.

In Zn(II)-malonate and Zn(II)-citrate complexes the best fit is always given for a unique Zn–O shell. Therefore, the hydration shell and the O´s of the carboxylate are at the same distance from the Zn(II) cation, at around 1.96 and 2.02 A, respectively. The coordination number of the first shell in both cases is noticeably lower than that of the hydrated Zn(II) being compatible with the contraction observed in the shell distances. In opposition to Zn(II)-acetate, in both cases, in the range of pH studied in this work, there are not signatures of complexated species other than Zn(II)-malonate and Zn(II)-citrate, in their respective solutions.

## Conclusions

The pH-dependent speciation and the structure of the Zn(II)-acetate complex have been studied in an aqueous medium by combining Raman and XAFS spectroscopy. The modification of the vibrational modes of the carboxyl ligand through the complexation with Zn(II) indicates the Zn(II)-acetate complex has weak bidentate coordination in solution. The νC-C and Ac-H bands have shown to be sensitive not only to acetic acid dissociation but also can differentiate the Zn complexated from the non-complexated acetate species. The XAFS shows clear structural differences in the Zn(II)-acetate with respect to the hydrated Zn(II). The measured coordination of the Zn(II)-acetate is close to 4 and the shell distance contracts by about 0.1 Å with respect to the hydrated Zn(II), presenting evidences of the formation of Zn(II)-acetate_2_ complex at pH 5.5. In such case, the oxygens of the carboxyl would occupy the first coordination shell and the solvation shell is probably expanded to 2.74 Å. However, the determination of the Zn(II)-acetate, malonate and citrate complexes structure and stability requires further investigation and numerical calculations.

### Supplementary Information

Below is the link to the electronic supplementary material.

Supplementary file1 (DOCX 4833 KB)

## Data Availability

Data will be made available upon reasonable request.
