# Supplementary material for: Structure and complexation mechanism of aqueous Zn(II)-acetate complex studied by XAFS and Raman spectroscopies
Source: Anal Sci. 2024 Apr 5;40(6):1193–201. doi: 10.1007/s44211-024-00549-z (PMC11126429; doi:10.1007/s44211-024-00549-z)
Supplement: Supplementary file 1 — Supplementary file1 (DOCX 4833 KB) [file 44211_2024_549_MOESM1_ESM.docx]

**Supplementary Information for Structure and complexation mechanism of aqueous Zn-acetate complex studied by XAFS and Raman spectroscopies.**

**Alvaro Munoz-Noval ^1, 2,*^, Kazuhiro Fukami^3^, Takuya Kuruma^2^, Shinjiro Hayakawa^2^**

^1^ Department of Materials Physics, Faculty of Physics, University Complutense of Madrid, Madrid, 28040, Spain

^2^ Department of Applied Chemistry, Graduate School of Engineering, Hiroshima University, Hiroshima 739-8527, Japan

^3^ Department of Materials Science and Engineering, Kyoto University, Kyoto 606-8501, Japan

^*^ Corresponding authors: alvaromunoznoval@ucm.es,

Contains:

Figure S1. Raman spectra of the Ac and Zn/ Ac solutions

Figure S2. Calculated speciation curves for a Zn 0.5 M/ AA 1.0 M solution in water at room temperature as a function of pH.

Figure S3. Calculated and experimental pH dependency of the Ac-H and νsCC bands in the AA and Zn(II)-acetate solutions.

Figure S4. Detail of the Raman spectra in the region of the Out of plane-plane OCO rocking/ OCO bending and O-H stretching in protonated acetate (Ac-H) bands.

Table S5. C-C stretching and Ac-H band quantification.

Figure S6. FT of the EXAFS spectra and fittings of Zn-acetate solutions with pH ranging between 2 and 5.5.

Table S7. Experimental values for the Raman frequency difference between the symmetric and asymmetric COO stretching modes (∆s-a ).

Raman spectra of the Ac and Zn/ Ac solutions


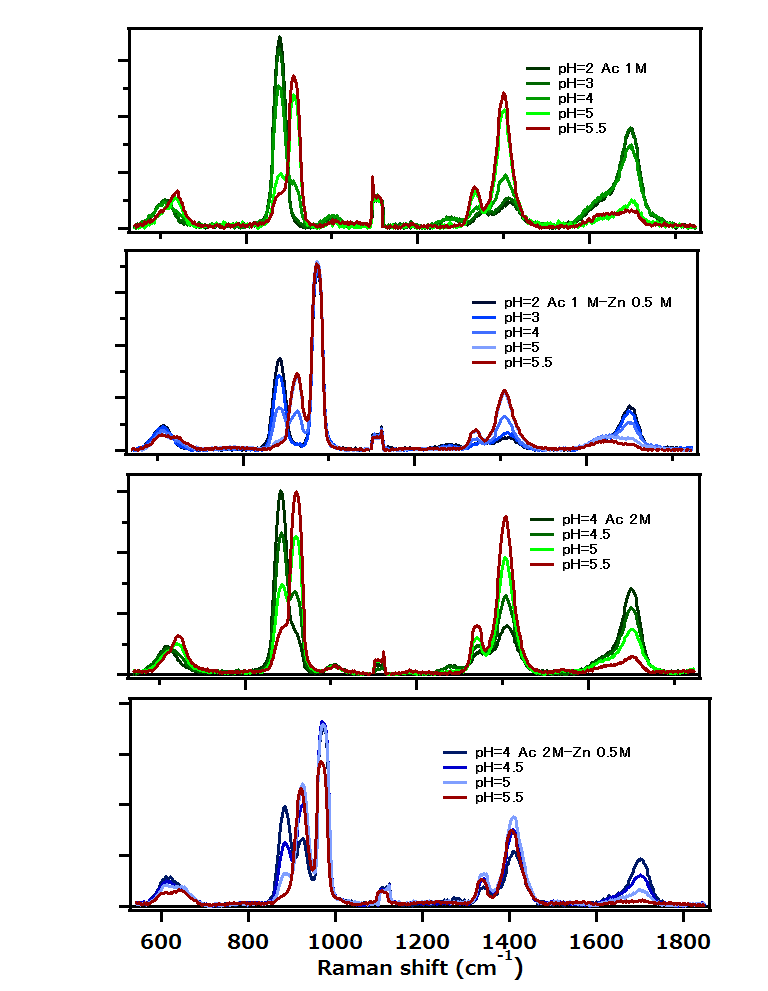


**Figure S1.** Raman spectra of the Ac and Zn-Ac solutions at different pH conditions.


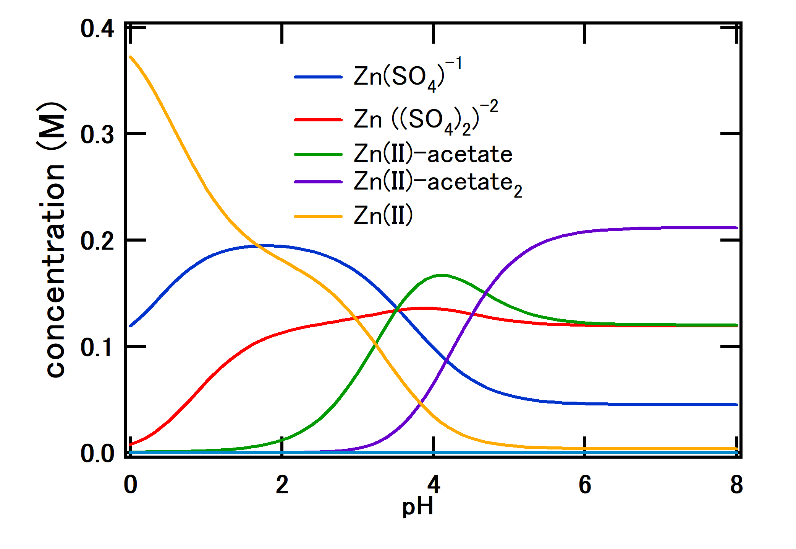


**Figure S2.** Calculated speciation curves for a Zn 0.5 M/ AA 1.0 M solution in water at room temperature as a function of pH.

**
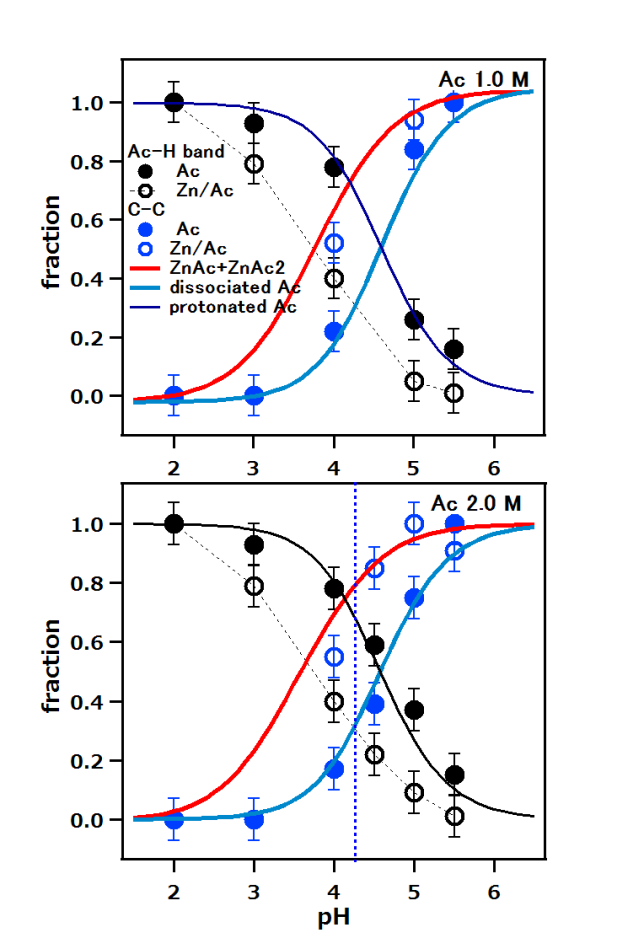
**

**pH**

**Figure S3.** pH dependency of the Ac-H and ν_s_CC bands in the AA and Zn(II)-acetate solutions (symbols) normalized and compared with the speciation curves of the acetate and Zn(II)-acetate. Values calculated below pH=4 are extrapolated from the ones obtained for 1.0 M AA solutions.

**
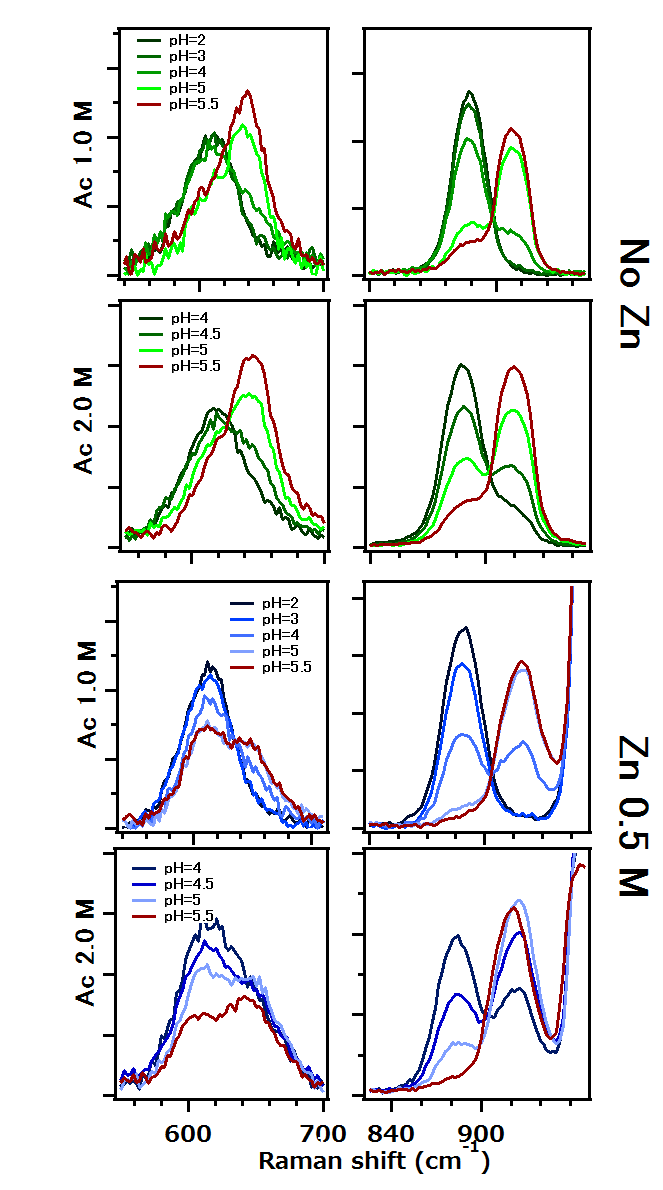
**

**Figure S4.** Detail of the Raman spectra in the region of the Out of plane-plane OCO rocking/ OCO bending (left columm) and O-H stretching in protonated acetate (Ac-H) bands (right column) for AA solutions 1.0 M and 2.0 M and Zn/AA solutions 0.5 M/1.0 M and 0.5 M/2.0 M that have been used to calculate the curves in Fig. SI.3.

**Table S5** C-C stretching and Ac-H band quantification.

|  | | Ac-H | | | C-C stretching | |
| --- | --- | --- | --- | --- | --- | --- |
| pH | | **No Zn** | **Zn 0.5 M** | | **No Zn** | **Zn 0.5 M** |
| Ac 1 M | **2** | **1.0** | | **1** | **0.00** | **0.00** |
|  | **3** | 0.93 | | 0.79 | 0.00 | 0.00 |
|  | **4** | 0.78 | | 0.40 | 0.22 | 0.52 |
|  | **5** | 0.26 | | 0.05 | 0.84 | 0.94 |
|  | **5.5** | 0.16 | | 0.01 | 1** | 1** |
| Ac 2 M | **4** | 0.78* | | 0.40* | 0.17* | 0.55* |
|  | **4.5** | 0.59* | | 0.22* | 0.39* | 0.85* |
|  | **5** | 0.37* | | 0.09* | 0.75* | 1** |
|  | **5.5** | 0.15* | | 0.01* | 1** | 0.91# |

**(*)using the pH= 4 1.0 M solution values as reference for normalization.**

**(**)using the intensity of the band at this condition as reference value for normalization of the lower pH conditions.**

**(#) large band shift to higher energies is observed.**

EXAFS fittings


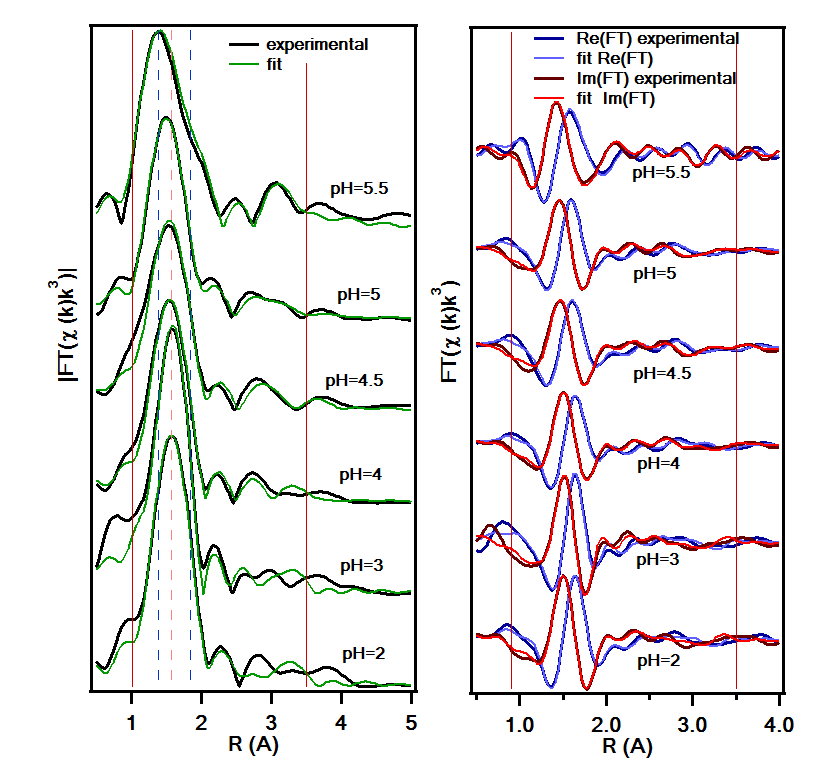


**Figure S6.** FT of the EXAFS spectra and fittings of Zn-acetate solutions with pH ranging between 2 and 5.5: a) module of the FT of the experimental data and fittings and b) same data and corresponding fits fits for the Real and Imaginary part of the FT.

**Table S7.** Experimental values for the Raman frequency difference between the symmetric and asymmetric COO stretching modes (*∆_s-a_* ) obtained for the AA and Zn/AA solutions with 2.0 M AA.

| solution | pH | *∆_s-a_ (cm^-1^)* |
| --- | --- | --- |
| **Ac 2.0 M** | **7** | **141.8** |
| **Zn-Ac 0.5M/2M** | **4** | ---- |
|  | **4.5** | 160.1 |
|  | **5** | 155.7 |
|  | **5.5** | 153.4 |
